# Moral, self-interested, and social motivation each predict compliance with social distancing rules: utilitarianism is an indirect positive predictor

**DOI:** 10.1186/s40359-023-01093-7

**Published:** 2023-03-29

**Authors:** Daniel B. Cohen, Lauren L. Saling, Eunro Lee, Anabella Zagura

**Affiliations:** 1 School of Social Work and Arts, Charles Sturt University, Wagga Wagga, NSW 2678 Australia; 2 School of Health and Biomedical Sciences, RMIT University, Bundoora, VIC 3083 Australia

**Keywords:** COVID-19, Social distancing rules, Compliance, Moral motivation, Self-interested motivation, Social motivation, Utilitarianism

## Abstract

**Background:**

Social distancing rules have proven to be essential in reducing the spread of COVID-19. However, we can optimise these rules if we identify factors which predict compliance. Thus, in this study we investigated whether compliance with distancing rules is predicted by whether an individual is motivated by moral, self-interested, or social reasons. We also investigated the impact of an individual’s utilitarian orientation both on compliance itself and on reasons for compliance.

**Methods:**

Our sample consisted of 301 participants recruited from four US states – California, Oregon, Mississippi, and Alabama – who completed an anonymous online survey. Six vignettes describing hypothetical social distancing rules were developed for the study. Participants indicated (i) how likely they were to violate each hypothetical distancing rule, (ii) how morally wrong violating each rule would be, (iii) how much risk of contracting COVID-19 they would tolerate in order to violate each rule, and (iv) how much social condemnation they would tolerate in order to violate each rule. Based on these responses, we gauged each participant’s overall degree of compliance with social distancing rules as well as the extent to which each participant’s compliance is motivated by moral, self-interested, and social reasons. We also measured other variables that could affect compliance including personality, level of religiosity, and inclination to engage in utilitarian reasoning. Multiple regression and exploratory structural equation modelling were used to determine predictors of compliance with social distancing rules.

**Results:**

We found that moral, self-interested, and social motivation each positively predicted compliance, with self-interested motivation being the strongest predictor. Furthermore, utilitarian orientation indirectly predicted compliance, with moral, self-interested, and social motivation as positive mediating factors. No controlled covariates (personality factors, religiosity, political orientation, or other background variables) predicted compliance.

**Conclusion:**

These findings have implications not only for the design of social distancing rules but also for efforts to ensure vaccine uptake. Governments need to consider how to harness moral, self-interested, and social motivation to promote compliance, perhaps by co-opting utilitarian reasoning, which positively influences these motivational forces.

## Background

In order to curtail the spread of COVID-19, many countries have instituted social distancing rules. However, compliance with these rules varies widely as a function of both individual differences and contextual factors. To maintain the efficacy of these preventative measures, it is important to identify factors that account for variations in compliance. This knowledge will allow governments to develop strategies to boost compliance. Even given the advent of COVID-19 vaccines, social distancing remains an important mechanism to reduce the spread of the virus because of inequitable distribution and limited accessibility of COVID-19 vaccines, particularly in developing countries [1] because of extensive vaccine hesitancy [2], and because emerging COVID-19 variants may not be reliably targeted by current vaccines.

We should expect various factors to explain variations in compliance with social distancing rules. Individual factors include age, personality, and political orientation, while contextual factors include stringency of legal enforcement, cultural attitudes, and population-density. For instance, Kleitman [3] found that non-compliant individuals scored lower on agreeableness, openness to experience, and adaptive coping strategies, and higher on extraversion, compared with compliant individuals. Younger adults are typically less compliant than older adults [4]. Differences along partisan lines have also been found, with fewer Republicans engaging in social distancing than Democrats [5].

In this study, we explored a novel factor: we investigated whether variation in compliance depends on differences in the kinds of reason which motivate individuals to comply with distancing rules. As we explain below, people may be motivated to comply with distancing rules by moral, self-interested, or social reasons. People also vary in their inclination to engage in utilitarian reasoning. We investigated whether these differences predict differences in compliance.

We also investigated individual factors, such as political orientation, age, and culture, which may predict the reasons which motivate people to comply with social distancing rules. In our investigation of cultural factors, we utilised the tight/loose distinction drawn by Gelfand [6]. Gelfand characterises ‘tight’ cultures as having clear, pervasive social norms which are strictly enforced and ‘loose’ cultures as having fewer, vaguer social norms, deviation from which is less strictly enforced. On the basis of these features, Gelfand [6] ranked U.S. states on the tight/loose continuum: the two loosest states, according to this ranking, are California and Oregon, while Mississippi and Alabama are the two tightest states. We thus investigated whether the reasons which motivate compliance with distancing rules varied across these four locations.

Understanding how an individual’s reasons for compliance affects their degree of compliance will allow governments to fine-tune future implementations of social distancing rules to maximise their effectiveness. This is important as COVID-19 returns in waves [7]. Even as our society overcomes COVID-19, future epidemics and pandemics may require similar social distancing rules and we can use the knowledge gained in the current crisis to inform strategies for their implementation. Our research has implications, for instance, regarding how distancing rules are advertised, what kinds of information are provided to motivate compliance, how this information is framed, and how distancing rules are enforced. Our research also has implications for health messaging more generally; for instance, in campaigns promoting vaccination. Of special importance is the question of the relative impact of moral and self-interested reasons in motivating people to engage in behaviours that prevent disease. This study makes a contribution towards answering this overarching question.

### Moral, self-interested, and social reasons

For the purposes of our investigation, we distinguished the following different reasons which may motivate a person to comply with social distancing rules.

**Moral reasons.** An individual practices social distancing for moral reasons when they are motivated by concern for others. There is evidence that moral reasoning plays a role in social distancing behaviour [8]. However, there are different ways in which the concern for others might motivate someone to comply with distancing rules. Broadly speaking, utilitarians – who seek to maximise overall happiness – will be motivated to practice social distancing because they believe that social contact risks harming other people [9]. In contrast, deontologists will be motivated by the belief that social distancing is required by valid moral rules [10].

The origin of valid moral rules is a matter of philosophical debate amongst deontologists: for instance, some deontologists (so-called ‘rule utilitarians’) think that moral rules prescribe patterns of behaviour which, if widely adopted, would maximise overall happiness [11]. However, all deontologists deny that the function of moral rules is simply to guide agents to maximise happiness: valid moral rules may thus prohibit harmless behaviour and they may even require harmful behaviour [12]. Thus, deontologists who believe that moral rules require social distancing may well be motivated to comply with distancing rules even when they judge the particular risks of their own non-compliance to be negligible. In contrast, it seems that utilitarians should be motivated to comply with distancing rules only to the extent that they expect that their own non-compliance will risk harming others. We thus investigated whether an individual’s inclination to endorse utilitarian reasoning predicts compliance with distancing rules.

**Self-interested reasons.** An individual’s self-interested reasons are determined by what they believe will maximise their own welfare [13]. Thus, self-interest may motivate someone to comply with social distancing rules if they fear catching COVID-19 and believe that social distancing measures will reduce their chance of infection. Self-interest could also motivate compliance with social distancing rules via the fear of punishment or social sanction for violation.

**Social reasons.** An individual practices social distancing for social reasons when they are motivated to respect a social rule requiring this behaviour. Social rules exist in virtue of normative expectations and norm-enforcement behaviours practiced within a community, e.g., praising and shunning [14]. Note that the motivation to respect a social rule does not depend on the belief that the rule has a good moral or self-interested justification – one is motivated to respect a social rule simply because one has internalised the rule as a norm for behaviour.

### Evidence suggesting the motivational significance of reasons for compliance

Just one other study, to our knowledge, has explored the role played by an individual’s reasons for compliance on the degree of their compliance with social distancing rules. Bicchieri [15] asked respondents to predict the behaviour of a hypothetical agent: respondents predicted that agents would have higher levels of compliance when compliance was high in the agent’s community and when compliance was expected within the community. This effect was moderated by respondents’ trust in science. This result suggests that people expect compliance with distancing rules to be influenced by social motivation. In contrast, in our study, we aimed to measure whether compliance is in fact influenced by social (as well as moral and self-interested) motivation.

There is evidence that underlying moral concern plays a role in the motivation to comply with social distancing rules: Maftei and Holman [16] found that morally disengaged participants were more likely to report lower compliance with COVID-19 social distancing measures. There is also relevant literature comparing the broad motivational impact of moral and self-interested reasons. For instance, Crockett [17] found that participants paid more to reduce others’ pain than their own, suggesting that people are more strongly motivated by moral reasons than by self-interested reasons.

### Message framing

A further relevant body of literature investigates the efficacy of message framing on compliance with social distancing rules. This research is relevant because effective message framing could function, in part, by triggering people to be morally, self-interestedly, or socially motivated to practice social distancing. Our research might thus illuminate why framing measures work when they do and also identify the limitations of framing measures.

Highlights of this literature to date are as follows: social distancing messages framed in terms of protecting a particular victim are more effective than messages framed in terms of ‘flattening the curve’ [18]; deontologically-framed messages predict increased hand washing and social distancing intentions whereas consequentialist-framed messages merely predict increased hand washing intentions [19]; and messages framed as ‘benefiting everyone’ are more convincing than messages framed as ‘benefitting you’ [20]. The research on message framing is, however, inconclusive: there remains much disagreement about how to optimally communicate social distancing rules to maximise compliance. However, we ought to be able to fine-tune message-framing more effectively once we establish which reasons for compliance have the greatest motivational impact.

### Aims and hypotheses of the present study

The aims of the present study were to determine:

1. whether an individual’s reasons for compliance with social distancing rules (specifically, whether they are motivated by moral, self-interested, or social reasons) affects their degree of compliance with social-distancing rules,
2. whether an individual’s inclination to endorse utilitarian reasoning affects their degree of compliance, and.
3. whether individual differences predict (a) an individual’s reasons for compliance with social distancing rules, and (b) the degree of their compliance.

The following hypotheses were tested:

- Motivation by social reasons will predict compliance with social distancing rules.

As the sensitivity to the behavioural expectations of others appears to be a core driver of human behaviour, we predicted that individuals who see social reason to comply with distancing rules will be more strongly motivated than those who don’t see social reason to comply.

b. Motivation by moral reasons will predict higher compliance with social distancing rules than motivation by self-interested reasons.

While we are sensitive to the behavioural expectations of others, irrespective of their basis, we are especially sensitive to expectations grounded in moral reasons. We thus predicted that individuals who see moral reason to comply with social distancing rules will be more strongly motivated than those who see only self-interested reason to comply.

c. Inclination to endorse utilitarian reasoning will predict lower compliance with social distancing rules.

Hypothesis (c) is justified given the nature of utilitarian reasoning. One has a utilitarian reason for complying with a social distancing rule only insofar as one’s non-compliance, under the circumstances, will cause harm to others; this suggests that utilitarian reasoning will predict a less consistent motivation to comply with distancing rules than deontological reasoning, which typically employs circumstance-invariant rules for behaviour. We thus predicted that the inclination to endorse utilitarian reasoning will negatively predict an individual’s degree of compliance with social distancing rules.

## Methods

### Participants

An a priori power analysis using GPower 3.1 with power = 0.8, effect size = 0.2 and 5 predictors, revealed that the minimum required sample size for this study was *N* = 70.

The study was advertised via Prolific (https://www.prolific.co/) and 310 participants were recruited between 23 February and 13 March 2021. Participants were paid the US dollar equivalent of £1 to complete an 8-minute survey. Data from 9 participants were excluded because they failed the attention check. The final sample consisted of 301 participants recruited from four US states: California, Oregon, Mississippi, and Alabama. There were 75 participants from each state, aside from Oregon from which there were 76 participants. These states were selected as they are the two ‘loosest’ and the two ‘tightest’ of US states, according to Gelfand [6]. We aimed to investigate whether residence in a tight or loose state would predict motivation for compliance or level of compliance. Sample demographics are summarised in Table 1.

**Table 1:** Demographics for the final sample

Demographic Variable	n	%
*Gender*		
Female	175	58.1
Male	123	40.9
Other	2	.7
Unspecified	1	.3
*Age*		
18–29	4	1.3
30–39	112	37.2
40–49	98	32.6
50–59	43	14.3
60–69	27	9.0
70–79	12	4.0
80+	5	1.6
*Highest Educational Level*		
Did not complete school	5	1.7
Year 12 or equivalent	45	15
Diploma	78	25.8
Bachelor’s Degree	124	41.2
Postgraduate Degree	49	16.3
*Religion*		
Christianity	129	42.9
Islam	3	1.0
Hinduism	1	.3
Buddhism	5	1.7
Judaism	6	2.0
None	128	42.5
Other	29	4.6
Prefer not to say	15	5.0

### Procedure

#### Ethical considerations

##### Ethics approval

was obtained from the relevant university Human Research Ethics Committee. Consent was implied by submission of the completed anonymous survey. In the unlikely event of distress as a result of participation in the study, contact details for appropriate support services were provided in the participant information statement. Participants completed the anonymous survey online via Qualtrics. All participants completed the measures outlined below.

### Measures

**Attention check.** The following question was asked twice in the course of the survey: “How effective do you think social distancing measures are to prevent the spread of COVID-19?” (0 = not at all effective and 100 = very effective). As one’s answer to this question should remain stable within the time-frame of the survey, any variation beyond 20 points was judged to be a function of inattention.

**Demographics.** Non-identifying demographic information was collected. This included age, gender, highest educational level, religion, religiosity, and political orientation. Participants were asked to rate their political orientation from Left to Right (0 = Left and 100 = Right).

**Beliefs about COVID-19.** Participants were asked to indicate their beliefs concerning (a) the average severity of COVID-19, (b) the severity of COVID-19 were they to personally contract it, (c) the effectiveness of social distancing measures to prevent the spread of COVID-19, (d) the likely degree of their own conformity with social distancing measures, and (e) the degree of conformity with social distancing measures of others in their location.

**Personality.** The Big Five Inventory–2 Extra-Short Form (BFI-2-XS) developed by Soto and John [21] was used to measure personality. It has three items for each of the five personality dimensions (extraversion, conscientiousness, openness to experience, agreeableness, and negative emotionality). Even though the BFI-2-XS is a short scale, it has good external validity. Internal consistency values are reported to be 71% of those of the full scale which is considered acceptable for a short scale; α values range from = 0.5 to 0.7 [21]. In the current study, α values ranged from 0.6 to 0.8.

**Degree of utilitarianism.** To measure the inclination to endorse utilitarian reasoning, we employed the Oxford Utilitarianism Scale (OUS) developed by Kahane et al. [22]. The OUS improves on earlier measures (e.g. [23]) which typically involve a single dimension of utilitarian thinking: the approval of instrumental harm in sacrificial dilemmas, where one must choose whether to kill some in order to save others. The OUS, in contrast, is a two-dimensional measure, which includes two subscales: Subscale 1 (Impartial Beneficence) consists of 5 items that measure the inclination to endorse actions promoting the greater good even at personal expense. A representative item says “If the only way to save another person’s life during an emergency is to sacrifice one’s own leg, then one is morally required to make this sacrifice”. Subscale 2 (Instrumental Harm) consists of 4 items that measure the willingness to endorse actions which harm others in order to maximise the greater good. A representative item says “It is morally right to harm an innocent person if harming them is a necessary means to helping several other innocent people”. All items are measured on a 7-point likert scale ranging from 1 = strongly disagree to 7 = strongly agree. Good construct validity of the scale is reported, but internal consistency measures are not reported [22]. In the present study, Cronbach’s α values were 0.78 for OUS Subscale 1 and 0.76 for OUS Subscale 2.

**Vignettes.** Six vignettes were developed for this study, each describing a hypothetical scenario in which someone violates a social distancing rule. These vignettes were based on social distancing measures which were implemented in different parts of the world in 2020 (vignettes are outlined in Appendix A). Vignettes were employed for the following reasons: (a) a participant’s imaginative engagement with specific scenarios should evoke a more reliable indication of their attitudes towards social distancing rules than one based on a general impression of distancing rules; (b) while participants vary in their personal experiences with distancing rules, vignettes enable us to compare participant attitudes towards a common set of hypothetical rules; and (c) by averaging a participant’s reactions towards a range of scenarios, we reduce the effect of their having biased attitudes towards particular kinds of rule.

**Measures attached to vignettes.** For each vignette, participants were asked to answer the following questions: (i) “How likely would you be to violate a social distancing rule like this given the opportunity and desire?” (0 = not at all likely, 100 = extremely likely); (ii) “How morally wrong do you think this rule violation is?” (0 = not at all wrong, 100 = extremely wrong); (iii). “Suppose that by violating this rule, you would risk contracting COVID-19. How much risk would you tolerate in order to violate the rule?” (0 = no risk, 100 = extremely high risk); and (iv) “Suppose that by violating this rule, you would incur some social condemnation. How much social condemnation would you tolerate in order to violate the rule?” (0 = none, 100 = extreme).

**Likelihood of violation.** We calculated the overall likelihood of a participant violating social distancing rules by computing the mean score for ‘likelihood of violation’ (their answer to question (i), above) across the six vignettes. As violation is inversely proportional with compliance, factors which negatively predict violation will positively predict compliance, and vice versa. Our interest ultimately concerns compliance with distancing rules – thus, while our results are presented as a function of violation, we frame our findings, in our discussion, as concerning predictors of compliance, not violation.

**Motivations for compliance.** We calculated a participant’s overall degree of moral motivation by averaging their six vignette-relative responses to question (ii): this indicates a participant’s average perception of the moral wrongness of violating a social distancing rule. We calculated a participant’s overall degree of self-interested motivation by averaging their six responses to question (iii): we took the average degree of risk of COVID-19 infection that a participant would tolerate in order to violate a social distancing rule to be inversely proportional with their degree of self-interested motivation. We calculated a participant’s overall degree of social motivation by averaging their six responses to question (iv): we took the average degree of social condemnation that a participant would tolerate in order to violate a social distancing rule to be inversely proportional with their degree of social motivation. (Note that the three sorts of motivation are not mutually exclusive. For instance, an agent may be motivated by both moral and self-interested reasons to promote the welfare of loved ones.)

## Results

Descriptive statistics for study variables are presented in Table 2. All variables violated the normal distribution assumption (both Kolmogorov-Smirnov & Shapiro-Wilk tests with *p* < .01). Hence, the bootstrapping technique was used in the analysis to deal with non-normality in the data [24].

**Table 2:** Descriptive statistics for study variables

Variable	M (SD)	Skewness	Kurtosis
Moral Motivation	55.21 (24.93)	− 0.31	− 0.61
Self-interested Motivation	22.52 (23.87)	1.43	1.66
Social Motivation	25.31 (25.64)	1.29	1.00
Extraversion	1.57 (3.80)	0.18	− 0.48
Agreeableness	15.10 (3.41)	− 0.65	0.08
Conscientiousness	13.75 (4.04)	− 0.17	− 0.68
Negative emotionality	12.44 (4.45)	− 0.18	− 0.68
Open-mindedness	15.64 (3.40)	− 0.51	− 0.28
Impartial beneficence	18.70 (6.06)	− 0.07	− 0.47
Instrumental harm	12.02 (4.80)	0.30	− 0.49
Political orientation	33.57 (28.48)	0.50	− 0.74
Religiosity	31.72 (34.39)	0.66	− 1.04
Efficacy social distancing	76.87 (22.87)	− 1.49	2.24
COVID-19 severity (general)	69.51 (23.83)	− 0.77	0.09
COVID-19 severity (personal)	6.29 (29.31)	− 0.32	− 0.99
Conformity (self)	87.35 (19.80)	− 2.51	7.02
Conformity (others)	53.57 (23.90)	− 0.20	− 0.81
Likelihood of violation	28.56 (24.36)	1.07	0.58

Note: Impartial beneficence = Oxford Utilitarianism Scale, Subscale 1; Instrumental harm = Oxford Utilitarianism Scale, Subscale 2; COVID-19 severity (general) = beliefs about the average severity of COVID-19; COVID-19 Severity (personal) = beliefs about the severity of COVID-19 were a participant to personally contract it; Conformity (self) = the degree of a participant’s own conformity with social distancing measures; Conformity (others) = beliefs about the degree of conformity with social distancing measures of others in a participant’s location

Correlations among study variables are presented in Table 3.

**Table 3:** Correlations among study variables

Variables	1	2	3	4	5	6	7	8	9	10	11	12	13	14	15	16	17	18	19
1.Moral motivation																			
2. Self-interested motivation	**0.41**																		
3. Social motivation	**0.40**	**0.82**																	
4. Age	− 0.06	0.04	− 0.06																
5. Extraversion	− 0.06	**− 0.23**	**− 0.23**	0.05															
6. Agreeableness	− 0.03	**0.17**	**0.17**	**0.13**	0.00														
7. Conscientiousness	**− 0.17**	**− 0.22**	**− 0.25**	**0.24**	**0.28**	**0.32**													
8. Negative emotionality	**0.23**	**0.20**	**0.21**	**− 0.20**	**− 0.32**	**− 0.23**	**− 0.52**												
9. Open-mindedness	**0.12**	**0.17**	0.08	0.02	**0.13**	**0.19**	0.07	− 0.01											
10. Impartial beneficence	**0.30**	**0.20**	**0.22**	− 0.08	**− 0.12**	**0.29**	− 0.05	**0.18**	**0.12**										
11. Instrumental harm	− 0.04	− 0.06	− 0.03	**− 0.18**	0.03	**− 0.17**	− 0.08	**0.14**	**− 0.12**	0.10									
12. Political orientation	**− 0.38**	**− 0.40**	**− 0.40**	**0.16**	0.09	− 0.06	**0.23**	**− 0.29**	**− 0.22**	**− 0.22**	− 0.02								
13. Tight/ Loose	− 0.02	0.06	0.04	**0.18**	− 0.03	0.09	**0.16**	− 0.06	0.06	− 0.06	− 0.02	**0.12**							
14. Religiosity	**− 0.14**	**− 0.22**	**− 0.22**	0.08	0.10	**0.12**	**0.13**	**− 0.20**	− 0.05	− 0.01	**− 0.13**	**0.47**	**0.25**						
15. Efficacy social distancing	**0.53**	**0.53**	**0.45**	− 0.01	**− 0.12**	**0.21**	− 0.08	**0.12**	**0.13**	**0.30**	0.00	**− 0.38**	0.03	− 0.09					
16. COVID-19 severity (General)	**0.54**	**0.45**	**0.39**	− 0.06	− 0.10	**0.13**	− 0.09	**0.24**	**0.13**	**0.28**	− 0.02	**− 0.35**	0.01	− 0.07	**0.60**				
17. COVID-19 severity (Personal)	**0.37**	**0.30**	**0.19**	**0.19**	− 0.07	0.05	**− .0**2	**0.23**	**0.11**	**0.18**	− 0.09	**− 0.22**	0.11	0.01	**0.38**	**0.71**			
18. Conformity (self)	**0.53**	**0.62**	**0.53**	**− .0**4	**− 0.15**	**0.20**	**− 0.12**	**0.19**	**0.22**	**0.25**	0.01	**− 0.35**	− 0.01	− 0.10	**0.74**	**0.54**	**0.36**		
19. Conformity (others)	− 0.01	− 0.03	− 0.05	**0.17**	0.02	**0.15**	0.09	**− 0.20**	− 0.03	0.02	− 0.05	0.07	**− 0.31**	0.04	**0.14**	0.09	0.10	**0.19**	
20. Likelihood of violation	**− 0.55**	**− 0.79**	**− 0.72**	0.05	**0.15**	**− 0.15**	**0.21**	**− 0.23**	− 0.11	**− 0.28**	0.01	**0.43**	0.02	**0.21**	**− 0.53**	**− 0.45**	**− 0.30**	**− 0.61**	0.04

Note: *n* = 301. Significant correlations are in bold (*p* < .05 or *p* < .01)

The following variables were significantly positively correlated with each other: moral, self-interested, and social motivation and impartial beneficence. These variables were also each significantly negatively correlated with the likelihood of violation of social distancing rules. Political orientation correlated negatively with moral, self-interested, and social motivation. Given that political orientation ranged from Left = 0 to Right = 100, this negative correlation demonstrates that left-leaning political orientation is associated with higher moral, self-interested, and social motivation.

### Predictors of the likelihood of violating social distancing rules

A multiple regression analysis was undertaken to determine predictors of the likelihood of violation of social distancing rules. Predictors were moral motivation, self-interested motivation, social motivation, and two utilitarian factors (impartial beneficence and instrumental harm), with the following covariates: political orientation, religiosity, residence in a tight/loose state, five personality factors, and demographic variables of age, education, and gender. For statistical stability, 3 participants (who nominated a gender other than male or female) were excluded for all regression analyses (*n* = 298).

The overall model was significant, *F* (15, 282) = 45.68, *p* < .001, *R*^*2*^ = 0.71. Moral, self-interested, and social motivation each negatively predicted the likelihood of violation. The standardised coefficients showed that self-interested motivation had the largest effect – more than double the coefficient of moral motivation – while moral motivation had a coefficient almost double that of social motivation. No other variables or covariates were significant in explaining variance in the likelihood of violation (Table 4).

**Table 4:** Regression 1: Unstandardised (*b*) and standardised (β) coefficients for predictors of the likelihood of social distancing rule violation

Predictor Variable		Unstandardized Coefficients		Standardized Coefficients	*p*	95% Confidence Interval for *b*	
		*b*	SE	β		Lower Bound	Upper Bound
	Age	0.57	0.69	0.03	0.41	− 0.78	1.92
	Gender	− 0.09	1.73	0.00	0.96	− 3.50	3.33
	Education level	− 1.34	0.82	− 0.05	0.11	− 2.96	0.28
	Religiosity	− 0.01	0.03	− 0.01	0.71	− 0.06	0.04
	Political orientation	0.04	0.04	0.05	0.25	− 0.03	0.11
	Tight/Loose	1.96	1.69	0.04	0.25	− 1.27	5.29
	Extraversion	− 0.27	0.23	− 0.04	0.25	− 0.73	0.19
	Agreeableness	− 0.21	0.28	− 0.03	0.46	− 0.75	0.34
	Conscientiousness	− 0.08	0.25	− 0.01	0.76	− 0.58	0.42
	Negative emotionality	− 0.20	0.23	− 0.04	0.39	− 0.66	0.26
	Open-mindedness	0.39	0.25	0.05	0.12	− 0.10	0.88
	Impartial beneficence	− 0.17	0.15	− 0.04	0.25	− 0.47	0.12
	Instrumental harm	− 0.14	0.18	− 0.03	0.42	− 0.49	0.21
	**Moral motivation**	**− 0.23**	**0.04**	**− 0.23**	**< 0.001**	**− 0.30**	**− 0.15**
	**Self-interested motivation**	**− 0.58**	**0.06**	**− 0.58**	**< 0.001**	**− 0.70**	**− 0.46**
	**Social motivation**	**− 0.12**	**0.06**	**− 0.13**	**0.03**	**− 0.23**	**0.01**

Note: *n* = 298. Gender: male = 0, female = 1; Level of Religiosity: 0-100;. Political orientation: Left = 0 to Right = 10. Loose culture states = 1, Tight culture states = 2. Significant results are in bold

We also ran two analyses to investigate whether the relationship between self-interested motivation and the likelihood of violation is mediated by the perceived severity of COVID-19. Our first analysis found that perceived COVID-19 severity for people in general mediated the relationship between self-interested motivation and likelihood of violation (Indirect effect = − 0.0542, CI [-0.1097, − 0.0103]). However, our second analysis found no similar effect for perceived COVID-19 severity for the individual.

### Predictors of motivation

As we aimed to investigate whether individual differences predict an individual’s motivations for compliance with social distancing rules, three further multiple regression analyses were undertaken. Each analysis employed the following 13 predictors: two utilitarian factors (impartial beneficence and instrumental harm), political orientation, residence in a tight/loose state, religiosity, five personality factors, age, gender, and education level. Significant coefficients of the three regression analysis models are presented in Table 5. Notably, most covariates were not significant.

**Regression 2: Moral motivation.** For this regression, social and self-interested motivation were included as predictors of moral motivation in addition to the 13 predictors listed above. The overall model was significant, *F* (15, 282) = 7.41, *p* < .001, *R*^*2*^ = 0.28. Impartial beneficence and self-interested motivation positively predicted moral motivation while political orientation negatively predicted moral motivation. Given that political orientation ranged from Left = 0 to Right = 100, this negative correlation demonstrates that a left-leaning political orientation predicted higher moral motivation. There were no other significant predictors.

**Regression 3: Self-interested motivation.** For this regression, social and moral motivation were included as predictors of self-interested motivation in addition to the 13 predictors listed above. The overall model was significant, *F* (15, 282) = 45.68, *p* < .001, *R*^*2*^ = 0.71. Social and moral motivation, age, and open-mindedness positively predicted self-interested motivation while no other variables showed significant coefficients.

**Regression 4: Social motivation.** For this regression, self-interested and moral motivation were included as predictors of social motivation in addition to the 13 predictors listed above. The overall model was significant, *F* (15, 282) = 45.05, *p* < .001, *R*^*2*^ = 0.71. Age and open-mindedness negatively predicted social motivation while self-interested motivation positively predicted social motivation.

**Table 5:** Regressions 2, 3, & 4: Unstandardised (*b*) and standardised (β) coefficients for the significant predictors of moral, self-interested, and social motivation

			Unstandardized Coefficients		Standardized Coefficients				95% Confidence Interval for *b*	
**Outcome**	**Predictor**		*b*	SE		β	*p*	Lower Bound		Upper Bound
Moral motivation		Impartial beneficence	0.89	0.23		0.22	< 0.001	0.43		1.35
		Political orientation	− 0.19	0.06		− 0.22	0.001	− 0.30		− 0.08
		Self-interested motivation	0.21	0.10		0.20	0.03	0.02		0.40
Self-interested motivation		Age	1.99	0.67		0.10	0.003	0.67		3.31
		Open-mindedness	0.68	0.24		0.09	0.01	0.20		1.16
		Social motivation	0.70	0.04		0.74	<0.001	0.62		0.77
		Moral motivation	0.08	0.04		0.08	0.03	0.01		0.15
Social motivation		Age	-1.77	0.72		− 0.09	0.02	-3.20		− 0.35
		Open-mindedness	− 0.64	0.26		− 0.08	0.02	-1.16		− 0.12
		Self-interested motivation	0.80	0.04		0.75	< 0.001	0.72		0.89

Note: *n* = 298. Only significant coefficients are presented due to space limitations. Full results are available on request

### Does moral motivation mediate a relationship between utilitarian reasoning and the violation of social distancing rules?

At this point in the analysis, we had reason to suspect that moral motivation might mediate a relationship between impartial beneficence and the likelihood of violating social distancing rules. This was suggested by the following findings: (a) moral motivation negatively predicts violation (as per Regression 1); (b) impartial beneficence positively predicts moral motivation (as per Regression 2); and (c) impartial beneficence correlates negatively with (but does not itself predict) violation. (Note that impartial beneficence didn’t predict social or self-interested motivation.) Together, these findings suggest that impartial beneficence may be a distally relevant factor in predicting violation. Thus, in an exploratory analysis, we used Structural Equation Modelling (SEM) to test whether moral motivation mediates a relationship between impartial beneficence and violation. We also tested whether self-interested and social motivation play mediating roles.

Our first SEM included all study variables and individual control variables. Personality variables were omitted due to sample size limitations but were tested in later models with a reduced number of covariates. Strategically, later SEMs included a reduced set of control variables to maximise statistical power and stability given the sample size limitations.

**Fig. 1:**
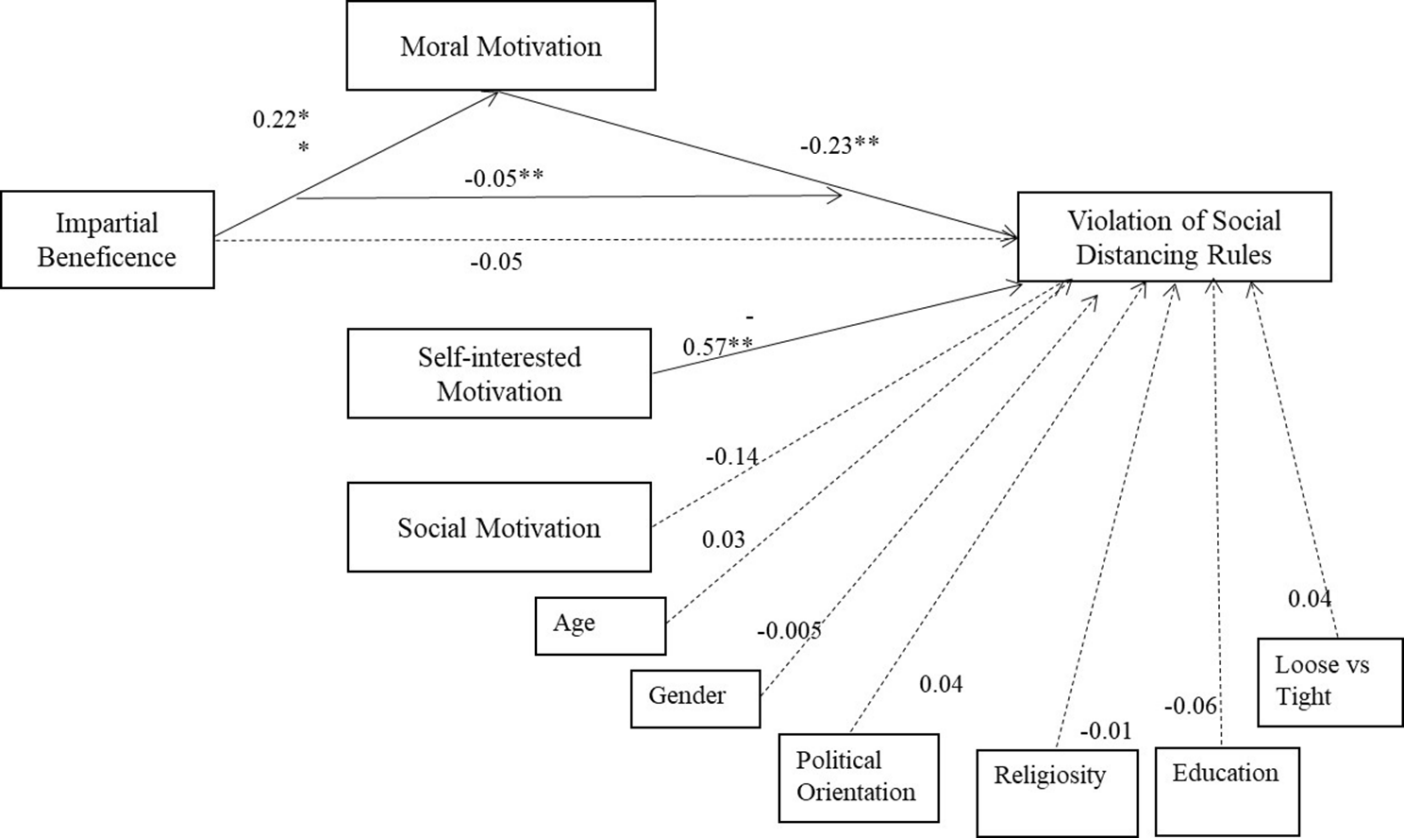
Structural equation model 1. Standardised coefficients (for model with all covariates except personality variables) of the full mediation effect of moral motivation on the impact of impartial beneficence on the likelihood of violation of social distancing rules. Note: n = 298. Correlations and error terms are omitted for visual simplicity.

**SEM 1: Full model with all covariates (except the personality variables).** For statistical stability, 3 participants (who nominated a gender other than male or female) were excluded for SEM 1 (this was the only SEM that included gender as a covariate). For this SEM, all study variables were included in attempting to explain the likelihood of violating social distancing rules, while moral motivation was examined as a mediator of impartial beneficence (Fig. 1). Although the model fits the data (χ^2^(31, *n* = 298) = 81.94, *p* < .001; NFI = 0.92; CFI = 0.95; RMSEA = 0.07), with 69% of the variance in the likelihood of violating social distancing rules explained by the predictors (*R*^*2*^ = 0.69), the results were deemed unstable due to the large number of estimated parameters (*k* = 46) for the given sample size. The model demonstrates that moral motivation mediates the impact of impartial beneficence on the likelihood of violating social distancing rules. Specifically, one standard deviation (SD) higher impartial beneficence is associated with 0.22 higher SD moral motivation which, in turn, is associated with 0.23 SD lower likelihood of violation suggesting an indirect effect of 0.05 SD lower likelihood of violation. One SD higher self-interested motivation is associated with 0.57 SD lower likelihood of violating social distancing rules. No coefficients of other variables are significant in the model.

**Fig. 2:**
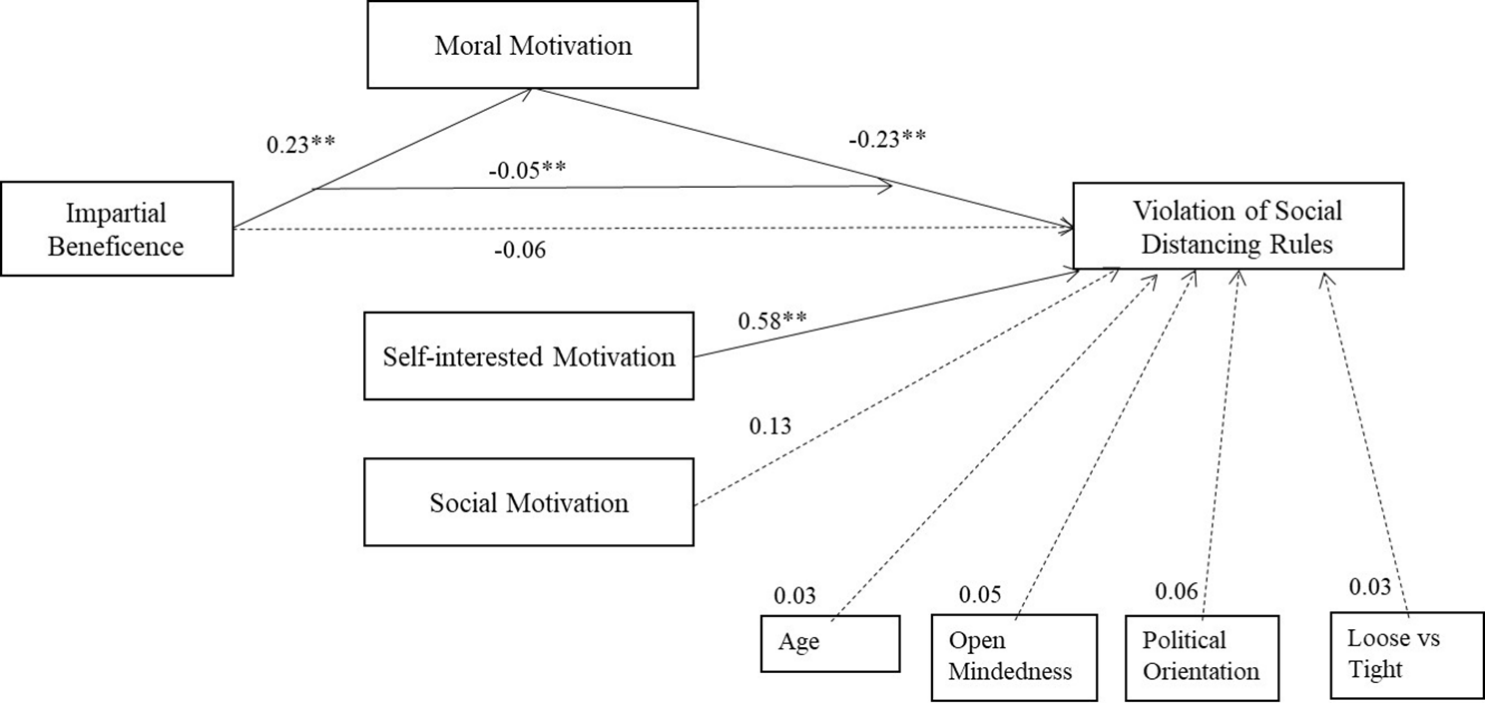
Structural equation model 2. Standardised coefficients (for reduced model with selected covariates) of the full mediation effect of moral motivation on the impact of impartial beneficence on the likelihood of violation of social distancing rules. Note: Correlations and error terms are omitted for visual simplicity. Model fit: χ^2^(15, *n* = 301) = 46.48, *p* < .001; NFI = 0.95; CFI = 0.96; RMSEA = 0.08. *R*^*2*^ = 0.70 (the likelihood of violation of social distancing rules)

**SEM 2: Reduced model with selected control variables.** Accommodating the sample size limitation, a reduced number of variables were included in our second SEM. The two significant covariates in the regression model – age and open-mindedness – were included while political orientation and the cultural variable of loose vs. tight states were controlled for considering the importance of these variables in previous studies. The model, which is presented in Fig. 2, again demonstrates that moral motivation mediates the impact of impartial beneficence on the likelihood of violating social distancing. The analysis shows that a higher degree of impartial beneficence is positively associated with greater moral motivation which, in turn, is negatively associated with the likelihood of violation. Thus, the analysis shows that impartial beneficence has an indirect effect on violation, via moral motivation. Self-interested motivation was also negatively associated with the likelihood of violation. Neither social motivation nor any other covariates significantly explain the variance in the likelihood of violation.

**Fig. 3:**
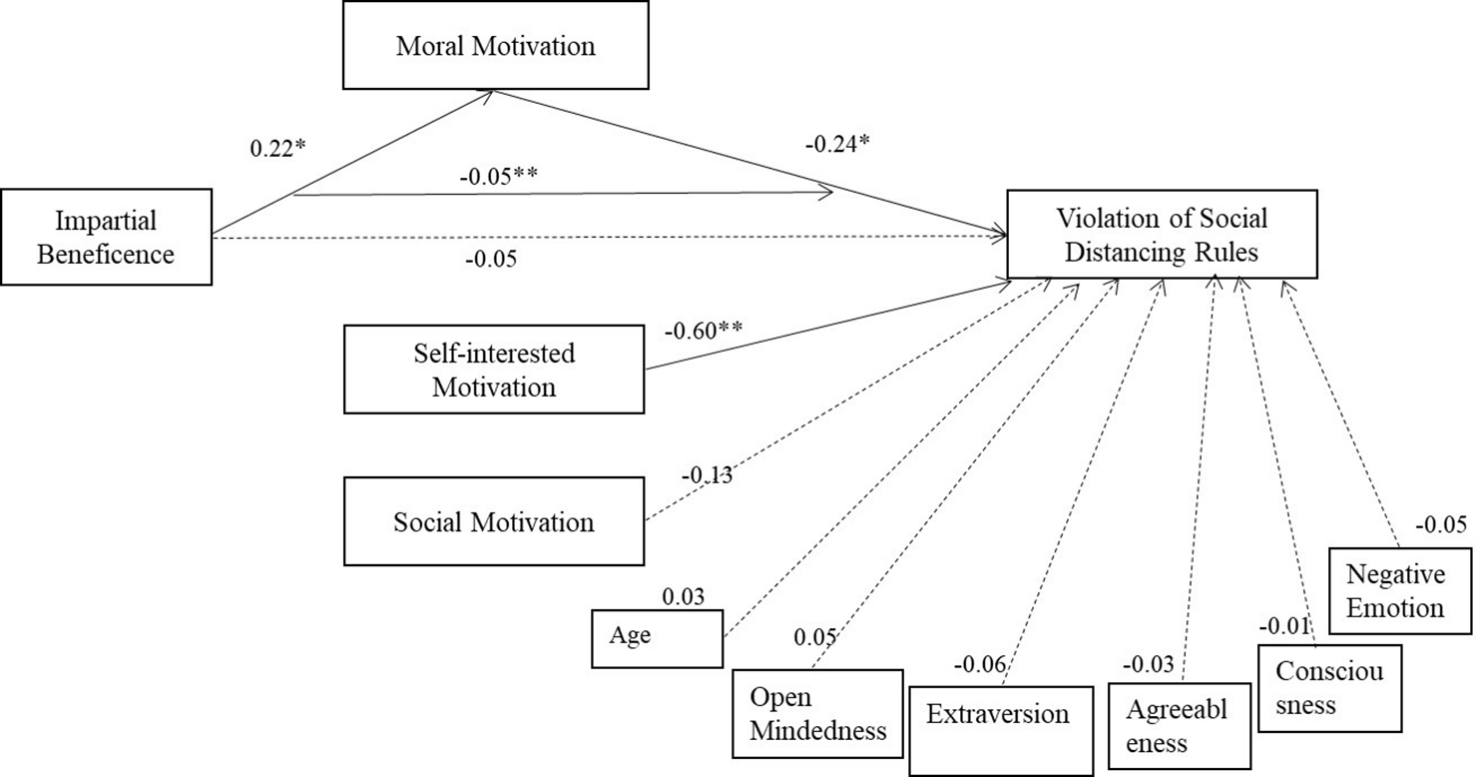
Structural equation model 3. Standardised coefficients (for a reduced model with selected covariates and personality variables) of the full mediation effect of moral motivation on the impact of impartial beneficence on the likelihood of violation of social distancing rules. Note: Correlations and error terms are omitted for visual simplicity. Model fit: χ^2^(27, *n* = 301) = 90.32, *p* < .001; NFI = 0.92; CFI = 0.94; RMSEA = 0.09. *R*^*2*^ = 0.69 (the likelihood of violation of social distancing rules)

**SEM 3: Reduced model with selected control variables and five personality factors.** Figure 3 presents a SEM with selected covariates guided by regression models to test the role played by personality in explaining the likelihood of violating social distancing rules. No personality variables or control variables are significant while the full mediation is significant, consistent with previous models.

**Fig. 4:**
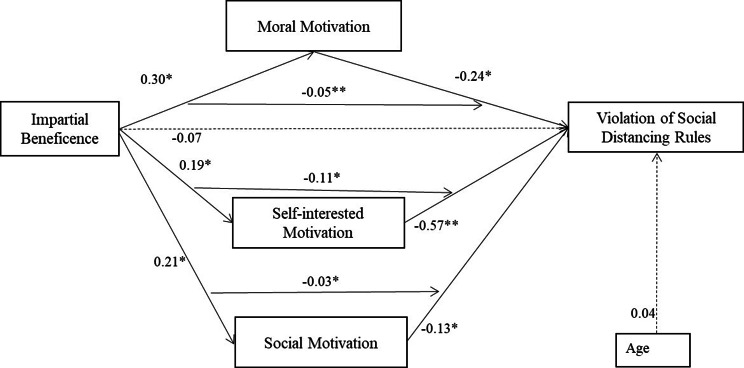
Structural Equation Model 4. Standardised Coefficients (for a reduced model with selected control variables) of the parallel mediation effect of three motivation variables on the impact of impartial beneficence on the likelihood of violation of social distancing rules. Note: Correlations and error terms are omitted for visual simplicity. Model fit: χ^2^(2, *n* = 301) = 1.81, *p* = .404; NFI = 0.99; CFI = 0.99; RMSEA = 0.00. *R*^*2*^ = 0.70 (the likelihood of violation of social distancing rules)

**SEM 4: Reduced model (with selected control variables) testing three parallel mediations by the three motivation variables.** Figure 4 presents a SEM to exploratively test the mediating roles of the three motivation variables on the impact of impartial beneficence on the likelihood of violation of social distancing rules. Age was added as a covariate (guided by the findings of Regression model 3). Note that SEM models 1–3 only tested the mediating effect of moral motivation. The model shows that impartial beneficence predicts each of the three motivation variables, each of which, in turn, significantly explains the variance in the likelihood of violating social distancing rules (all three motivation variables negatively predicted violation).

## Discussion

Our key aim in this study was to investigate whether an individual’s motivation for complying with social distancing rules predicted their overall level of compliance. We found that moral, self-interested, and social motivation each positively predicted compliance with social distancing rules. Moreover, we found that all three motivations significantly positively mediated the impact of impartial beneficence on compliance. This was confirmed in the final structural equation model which is the most comprehensive investigation of the research questions. Self-interested motivation, followed by moral motivation, played the most significant role in explaining the impact of impartial beneficence on compliance with social distancing rules. The wide range of covariates on individual differences were not significant while the models explained around 70% of variance in the likelihood of compliance, suggesting large effect sizes.

### Motivations for compliance

Consistent with hypothesis (a) (‘motivation by social reasons will predict compliance with social distancing rules’) we found that the higher one’s social motivation, the higher one’s compliance. However, social motivation was the weakest predictor of compliance, compared with moral and self-interested motivation. This finding suggests that the mere internalisation of a social rule has less motivational power than is predicted by the theoretical literature. If vindicated, this finding could warrant a revision of theoretically-motivated claims about the power of social rules that lack moral content.

Contrary to hypothesis (b) (‘motivation by moral reasons will predict higher compliance with social distancing rules than motivation by self-interested reasons’) we found that self-interested motivation was the stronger predictor of compliance. This finding is in tension with recent work in behavioural economics that emphasizes the role of moral motivation, rather than self-interest, in individual decision making [25].

Contrary to hypothesis (c) (‘inclination to endorse utilitarian reasoning will predict lower compliance with social distancing rules’), we found that utilitarian reasoning (specifically, impartial beneficence, as measured by the second OUS subscale) indirectly predicted higher compliance, where this relation was positively mediated by moral, self-interested, and social motivation. This finding is surprising because utilitarianism entails only a conditional need to comply with social distancing rules reflecting the specific risk posed by one’s own behaviour. However, the finding may reflect an implicit acceptance, amongst utilitarians, of ‘indirect’ reasons to adopt simple rules for behaviour (such as universal mask-wearing) in an approach that eschews a case-by-case calculation of likely outcomes [26].

A possible explanation for the mediation of utilitarian reasoning by moral motivation in its prediction of compliance with social distancing rules is that utilitarian reasoning is more likely than non-utilitarian reasoning to lead people to the conclusion that it is morally wrong to violate social distancing rules (see [27] for an exploration of this hypothesis).

The mediation of utilitarian reasoning by self-interested and social motivation in its prediction of compliance suggests that moral judgements may sometimes harness non-moral sources of motivation. While morality and self-interest are often construed as essentially in conflict (requiring inconsistent patterns of behaviour), this finding suggests that the two sorts of motivation can work together. In particular, the finding suggests that utilitarian moral reasoning may harness self-interested and social forces in producing motivation. An upshot is that moral motivation doesn’t necessarily require suppression of self-interest. Alternatively, this finding could indicate that moral, self-interested, and social motivation draw on similar cognitive resources; as a result, when prompted by utilitarian reasoning, moral motivation might activate social and self-interested motivation as a side-effect.

## Conclusion

We found that moral, self-interested, and social motivation each predicted compliance with social distancing rules. A practical implication of this finding is to raise doubts about the likely efficacy of campaigns designed to increase social distancing compliance which model agents as primarily self-interested actors who respond best to incentives that appeal to their welfare. Successful efforts to encourage social distancing will need to appeal to an individual’s moral and social motives as well.

The exact form that such appeals should take is a topic for further investigation, but given the inconsistent effects noted in the extant framing literature, it seems unlikely that the mere framing of social distancing rules in moral terms is sufficient to harness moral motivation. We also found that utilitarian reasoning predicts compliance via the mediating effect of moral, self-interested and social motivation. Thus, utilitarian reasoning could, in particular, be harnessed in campaigns designed to motivate compliance with social distancing measures. Overall, our findings confirm our hypothesis that motivational factors play an important role in predicting compliance with public health measures. However, their interaction with other factors is complex and deserves further investigation.

## Limitations and directions for further research

- Our participants were drawn from the two ‘tightest’ and the two ‘loosest’ US states, as per the tight/loose continuum developed by Gelfand [6]. As Gelfand’s measure is novel, future research should draw participants from a wider selection of locations to more reliably test the impact of political culture on compliance with distancing measures,.
- Given their location, our participants may not have directly experienced social distancing measures like those described in the vignettes. It is possible that participants with direct relevant experience would differ significantly in their responses. This presents a further reason for sampling participants from a wider selection of locations in future research.
- In order to provide deeper insight into the contextual factors which determine motivation with distancing measures, future research may benefit by including a qualitative element.
- The present study does not explore the potential impact on compliance played by structural factors such as social class. Future research in this area should consider structural factors.

## Data Availability

The dataset supporting the conclusions of this article is available in the OSF repository, https://osf.io/ujvxz/?view_only=f94695dc758942c7a13c13f229d7ff4b.

## Appendix A: Vignettes of hypothetical violations of social distancing rules

- Vignette 1: “The mayor has required everyone to take their temperature before leaving home, and to stay home if it is above 37.5℃/99.5℉. Aiden doesn’t feel hot, and is running late this morning, so he chooses not take his temperature on this occasion.”
- Vignette 2: “Although the government has forbidden gatherings of more than 10 people, Francesca is planning a birthday party for her daughter, with a total of 16 people.”
- Vignette 3: “Miguel’s young children are really missing playing at the playground, which is closed to prevent the spread of COVID-19. One day, when no one else is around, Miguel lets his children play in the playground.”
- Vignette 4: “Sasha is a hairdresser and has been forced to close her salon due to social distancing measures. She continues to cut clients’ hair in her basement.”
- Vignette 5: “Matilda and Fabian have just returned from overseas and are subject to a 2-week quarantine period. However, on many nights, they secretly leave their accommodation to get pizza.”
- Vignette 6: “All cafe patrons are required to leave their contact details to facilitate contact tracing in case a COVID cluster emerges at the venue. However, Jet is reluctant to share his info and enters a fake name and number whenever he goes to a cafe.”
